# Supplementary material for: Fucoidan and Fucoxanthin Attenuate Hepatic Steatosis and Inflammation of NAFLD through Modulation of Leptin/Adiponectin Axis
Source: Mar Drugs. 2021 Mar 12;19(3):148. doi: 10.3390/md19030148 (PMC8001566; doi:10.3390/md19030148)
Supplement: Supplementary file 1 [file marinedrugs-19-00148-s001.pdf]

Supplementary Data

Supplementary methods

Alamar Blue cell viability assay

The protective effects of LMF-HSFx on PA-induced cytotoxicity on HepaRG cells were determined using a modified Alamar Blue colorimetric drug screening assay. In brief, appropriate amounts of cells (1,500/well) were seeded onto 96-well flat-bottomed plates in 200 μl medium. After a 24 h incubation, the cells were treated with different concentrations (0, 25 and 50 μg/ml) of LMF-HSFx in the presence or absence of PA (200 or 400 μg/ml, respectively) followed by incubation for 24 h. Alamar Blue® (Invitrogen, Mississauga, ON, Canada) was added (10% of final volume) to the treated cells, which were then incubated at 37 °C. Following an incubation time of 3 h, the absorbance of test and control wells was read at 544 nm excitation and 590 nm emission with a standard spectrophotometer (SpectraMax M2e, Molecular Devices, San Jose, CA, USA). A subtraction analysis of the dual wavelength was performed to obtain accurate measurements.

Terminal deoxynucleotidyl transferase dUTP nick end labeling (TUNEL) assay

The BrdU-TUNEL assay was performed as described in manual. The HepaRG cells were treated with various concentrations of LMF-HSFx in the presence or absence of PA for 24 h in an 8-well chamber slide (iBidi, Gräfelfing, Germany). At the end of the incubation, the medium was removed directly and wash the cells with PBS once. Following, 0.5 ml of 1% (w/v) paraformaldehyde in PBS was added and placed on ice for 15 min. After discarding the supernatant, we washed the cells twice with PBS and then added 0.1 ml of PBS. Then the suspension was gently

added to 0.5 ml of ice-cold 70% (v/v) ethanol. The slide was stored in a –20 ℃ freezer overnight before analysis. The supernatant was removed after centrifuge and 50 µl of DNA-labeling solution (containing 10 µl of reaction buffer, 0.75 µl of TdT enzyme, 8.0 µl of BrdUTP and 31.25 µl of dH_2O) was added and the slide was stood in a cell culture incubator for 1 h. After washing the cells twice with 0.5 ml of Rinse Buffer, 100 µl of antibody staining solution (containing 5.0 µL of the Alexa Fluor™ 488 dye–labeled anti-BrdU antibody and 95 µl of Rinse Buffer) was added and incubated for 30 min at room temperature in the dark. Finally, 0.5 ml of the DAPI/RNase A staining buffer was added and then imaged the cells at Excitation 358 nm and Emission 461 nm for DAPI and Excitation 490 nm and Emission 525 nm for Alexa Fluor™ 488 by a fluorescence microscope (ImageXpress® Micro XLS, Molecular Devices, San Jose, CA, USA), respectively. Data were represented as 200 cells with fragmented DNA in each field (objective, 20X) and calculated from five independent fields.

Caspase 3 activity analysis

The activity of caspase-3 was assessed using a fluorometric Caspase 3 assay kit (Abcam, Cambridge, UK) according to the manufacturer's protocol. The caspase-3 assay is based on detection of cleaved fluorescent DEVD-7-amino-4-trifluoromethyl coumarin (AFC). In brief, the pre-cooled cell lysate from treated HepaRG cells was mixed with reaction buffer containing the DEVD-AFC substrate and then incubated for 1-2 h at 37˚C in a 96-well plate. The fluorescence was measured with a fluorescence reader (SpectraMax M2e, Molecular Devices, San Jose, CA, USA) using an excitation wavelength of 405 nm and an emission wavelength of 538 nm. Fold increase in caspase-3 activity was determined by comparing the results for the treated groups with those of the untreated control and normalized to the respective protein concentration.

Supplementary results

LMF-HSFx protect hepatocytes against palmitic acid-induced cytotoxicity and DNA damage.

We investigated the effect of LMF-HSFx on palmitic acid-mediated hepatic lipotoxicity. The data revealed that palmitic acid (PA) significantly reduced the cell number and induced cell de-attachment after 24 h of treatment (Supplementary Figure S3A). PA induced the decrease in cell viability in a dose-depend manner, while LMF-HSFx obviously inhibited PA-induced cytotoxicity (Figure S3B). Furthermore, it was revealed that PA significantly induced DNA fragmentation in hepatocytes, while co-treated the cells with LMF-HSFx notably reduced PA-induced DNA damage (Supplementary Figure S3C and S3D).

LMF-HSFx ameliorate palmitic acid-induced cell cycle arrest

The effect of LMF-HSFx on cell apoptosis in PA-treated cells was then investigated. It was revealed that PA induced significant cell cycle arrest at the G2/M phase ($p<0.05$) for 24 h incubation (Supplementary Figure S4A and S4B). It was found that LMF-HSFx dramatically restored the cell cycle progression.

Caspase-3 inactivation by LMF-HSFx co-treatment in PA-induced apoptotic cell death.

Following the caspase activity in PA-treated hepatocytes and the effect of LMF-HSFx was investigated. As presented in Supplementary Figure S7, caspase-3 was significantly ($p<0.05$) activated in PA-treated hepatocytes in comparison with untreated control cells after 24 h of

incubation, while LMF-HSFx co-treatment significantly ($P<0.05$) reduced PA-induced caspases activation.

Supplementary Figure legends

Supplementary Figure S1. LMF-HSFx reduces the insulin resistance in preDM patients.

The HOMA ratio of preDM patients (5 patients in each group) at 0, 1st ,3th and 6th month during the treatment. The relative ratio of insulin to baseline (%) and insulin secretion index (beta cell function index) of preDM and nonDM patients at 0, 1st ,3th and 6th month during the treatment.

Supplementary Figure S2. LMF-HSFx reduces the fibrosis in patients with stiffness degree higher than mild fibrosis F1 stage.

The interleaved scatter plot graph demonstrated the LMF-HSFx reduces the stiffness degree of patients with stiffness degree higher than F1 stage at 1st, 3rd and 6th month (*$p<0.05$, **$p<0.01$, compared with placebo). Stiffness stage: F1 (mild fibrosis): up to 7, the orange line; F2-3 (moderate-severe fibrosis): up to 8.7, the blue line; F4 (cirrhosis): up to 10.3, the red line. F0 (normal): 6, the green line.

Supplementary Figure S3. LMF-HSFx restores PA-induced cell death and DNA fragmentation of HepRG cells.

(A) Morphology and (B) summarized bars depict the cell viability by Alamar Blue assay of HepRG cells in PA 200 or 400 μM with or without LMF-HSFx 25 or 50 μg, LMF-HSFx 25 or 50 μg and control groups, taking control group as 100% (***$p<0.001$, compared with control; ##$p<0.01$, compared with PA200; $$$ $p<0.001$, compared with PA400). (C) the cell images (DAPI, blue;

DNA fragments, green) and (D) summarized bars depict DNA fragmentation of HepRG cells. (***p<0.001, *p<0.05, compared with control; $ p<0.05, compared with PA400).

Supplementary Figure S4. LMF-HSFx ameliorate PA-induced cell cycle arrest

(A) the cell cycles distribution of HepRG cells in PA 200 μM, PA 400 μM, LMF-HSFx 25 μg, PA 200 μM plus LMF-HSFx 25 μg, PA 400 μM plus LMF-HSFx 25 μg and untreated groups were measured with flow cytometry. (B) representative quantitative chart depicts cell cycle phase presented as the mean ± SD. (***p<0.001, **p<0.01, compared with control; #p<0.05, compared with PA200; $$ p<0.01, compared with PA400).

Supplementary Figure S5. LMF-HSFx mixture reduces the PA-induced Caspase 3 activation

Caspase 3 activity of HepRG cells in PA 200 μM, PA 400 μM, LMF-HSFx 25 μg, PA 200 μM plus LMF-HSFx 25 μg, PA 400 μM plus LMF-HSFx 25 μg and untreated control groups, taking control group cells as 1fold. (***p<0.001, compared with control; $ p<0.05, compared with PA400)

Supplementary table 1 Baseline characteristics

Demo characteristics of both groups			
	LMF-HSFx (n=21)	Placebo (n=21)	p-value
Anthropometry			
Age (years)	55±12.5	59±10.5	0.23
Male	7 (38.9%)	11 (61.1%)	0.35
Female	14 (58.3%)	10 (41.7%)	
Body mass index (BMI)	28.7±3.9	27.5±4.0	0.31
Laboratory data			
FBS (mmol/L)	97.8±17.3	103.2±14.7	0.29
Insulin (μIU/mL)	10.3±4.1	11.1±5.1	0.58
Total Cholesterol (mg/dL)	190.3±39.4	198.9±26.6	0.41
HDL (mg/dL)	50.5±9.4	50.2±9.9	0.91
LDL (mg/dL)	119.3±30.9	125±20.5	0.49
Triglyceride (mg/dL)	161.7±80.0	163.4±75.7	0.99
Adiponectin (μg/ml)	5652±2433.3	6486±3842.7	0.68
Leptin (μg/ml)	21.8±12.4	24.1±10.6	0.54
UA (mg/dL)	5.9±1.2	5.6±0.8	0.36
Cr (mg/dL)	0.74±0.1	0.72±0.2	0.71
AST (U/L)	28.1±7.0	28.5±15.9	0.33
ALT (U/L)	42.2±17.2	33.5±21.7	0.16
Stiffness (E, kPa)	6.5±1.8	6.7±2.9	0.52
Steatosis (CAP, dB/m)	343.6±44.6	303.6±66.9	0.03

Supplementary table 1 The baseline characteristics of both groups were similar, only the steatosis (CAP) was worse in LMF-HSFx group.

Figure S1

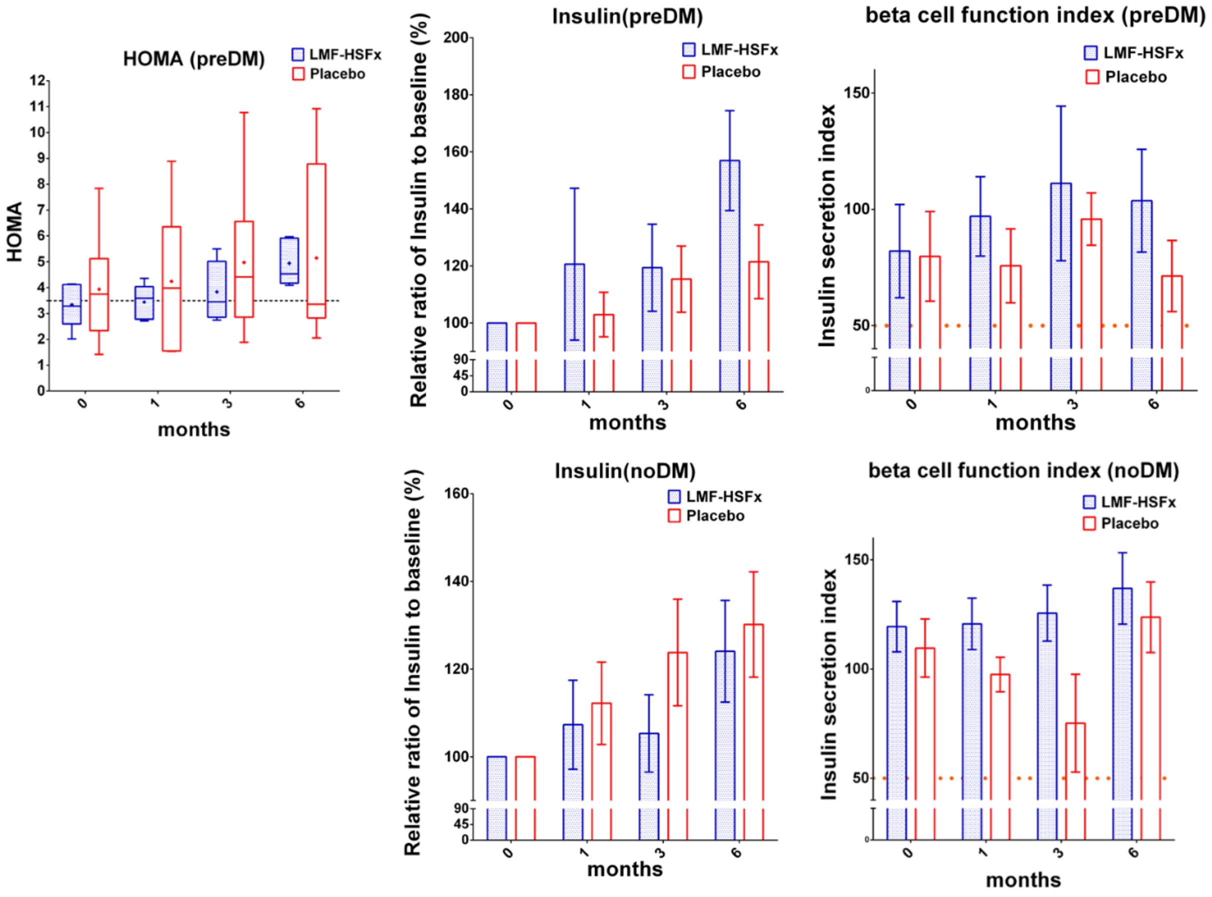
HOMA (preDM)
LMF-HSFx
Placebo
HOMA
months
Insulin(preDM)
Relative ratio of Insulin to baseline (%)
beta cell function index (preDM)
Insulin secretion index
Insulin(noDM)
beta cell function index (noDM)

Figure S2

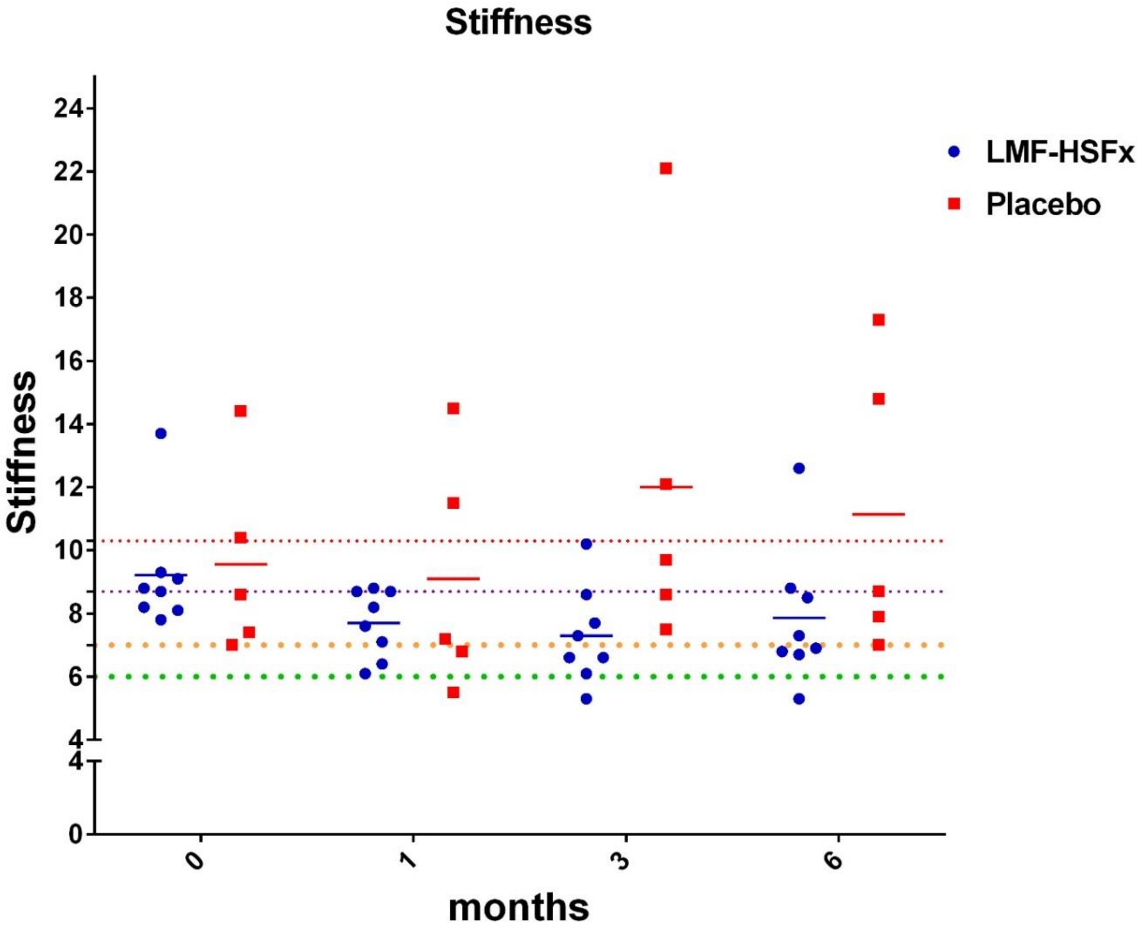
Stiffness
24
22
20
18
16
14
12
10
8
6
4
4
0
Stiffness
LMF-HSFx
Placebo
0
1
3
6
months

Figure S3

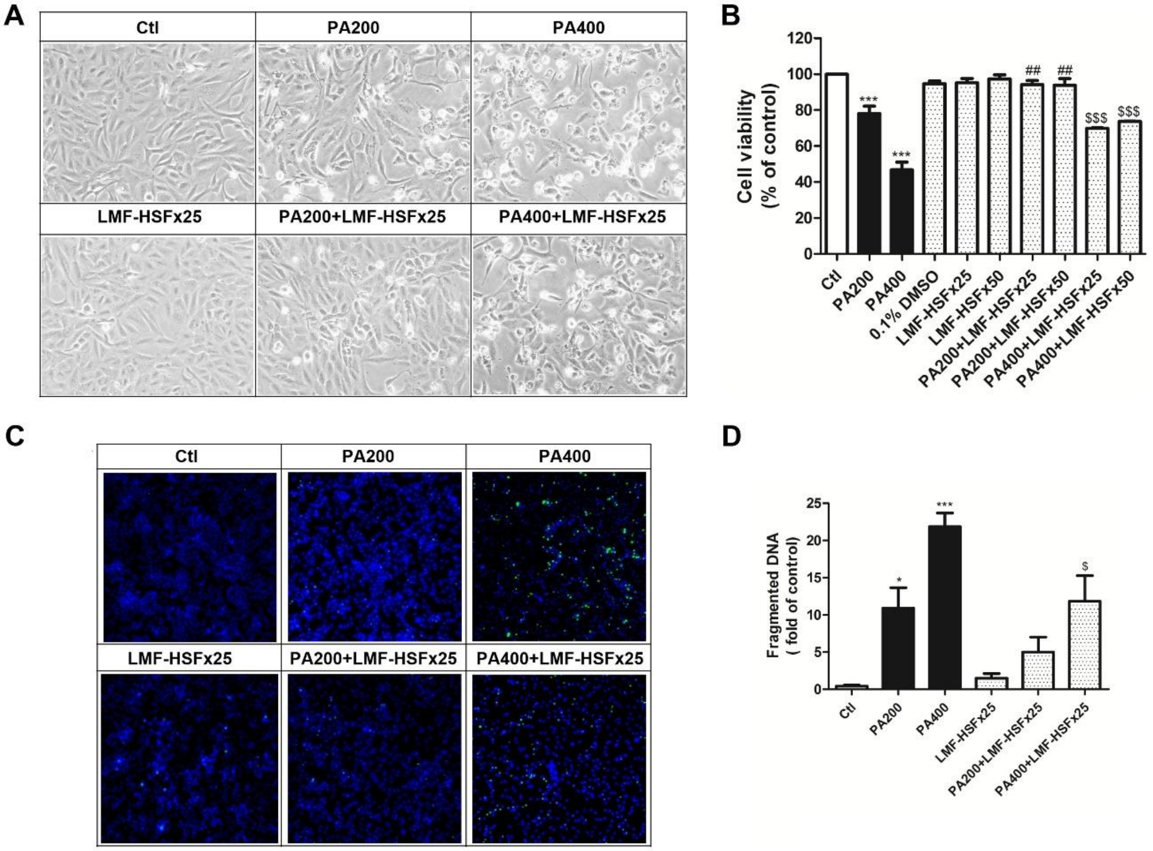
A
Ctl
PA200
PA400
LMF-HSFx25
PA200+LMF-HSFx25
PA400+LMF-HSFx25
B
Cell viability
(% of control)
120
100
80
60
40
20
0
***
***
##
##
$$$
$$$
Ctl
PA200
PA400
0.1% DMSO
LMF-HSFx25
LMF-HSFx50
PA200+LMF-HSFx25
PA200+LMF-HSFx50
PA400+LMF-HSFx25
PA400+LMF-HSFx50
C
Ctl
PA200
PA400
LMF-HSFx25
PA200+LMF-HSFx25
PA400+LMF-HSFx25
D
Fragmented DNA
(fold of control)
25
20
15
10
5
0
*
***
$
Ctl
PA200
PA400
LMF-HSFx25
PA200+LMF-HSFx25
PA400+LMF-HSFx25

Figure S4

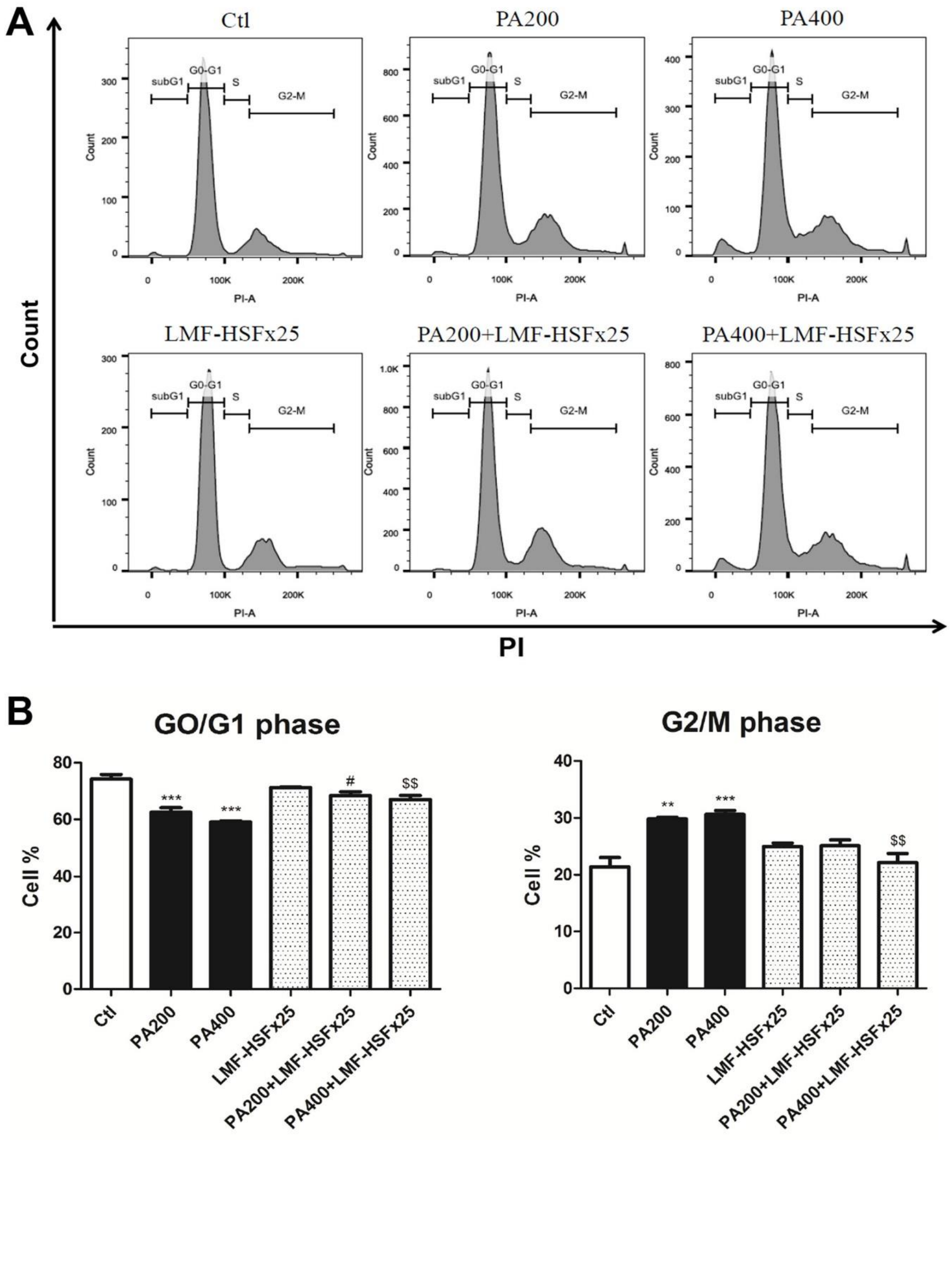
A
Ctl
PA200
PA400
LMF-HSFx25
PA200+LMF-HSFx25
PA400+LMF-HSFx25
subG1
G0-G1
S
G2-M
Count
PI-A
PI
B
GO/G1 phase
G2/M phase
Cell %
***
#
$$
**
Ctl
PA200
PA400
LMF-HSFx25
PA200+LMF-HSFx25
PA400+LMF-HSFx25

Figure S5

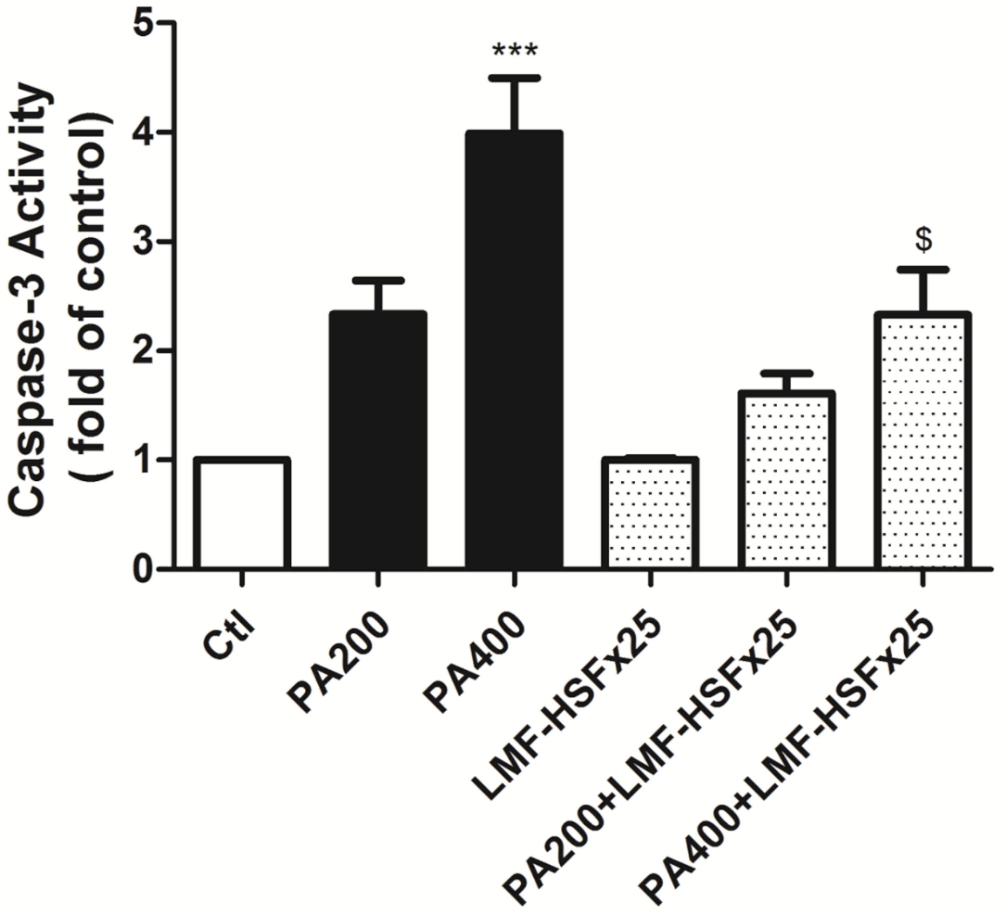
Caspase-3 Activity
(fold of control)
5
4
3
2
1
0
***
$
Ctl
PA200
PA400
LMF-HSFx25
PA200+LMF-HSFx25
PA400+LMF-HSFx25
